# Validity of administrative database detection of previously resolved hepatitis B virus in Japan

**DOI:** 10.1002/jmv.25540

**Published:** 2019-07-22

**Authors:** Shinobu Imai, Hayato Yamana, Norihiko Inoue, Manabu Akazawa, Hiromasa Horiguchi, Kiyohide Fushimi, Kiyoshi Migita, Hiroshi Yatsuhashi, Masaya Sugiyama, Masashi Mizokami

**Affiliations:** ^1^ Department of Clinical Data Management and Research, Clinical Research Center National Hospital Organization Headquarters Tokyo Japan; ^2^ Department of Drug Safety and Risk Management, School of Pharmacy Tokyo University of Pharmacy and Life Sciences Tokyo Japan; ^3^ Department of Health Services Research, Graduate School of Medicine The University of Tokyo Tokyo Japan; ^4^ Department of Health Policy and Informatics Tokyo Medical and Dental University Graduate School of Medicine Tokyo Japan; ^5^ Department of Public Health and Epidemiology Meiji Pharmaceutical University Tokyo Japan; ^6^ Department of Rheumatology Fukushima Medical University School of Medicine Fukushima Japan; ^7^ National Hospital Organization, Nagasaki Medical Center Omura Japan; ^8^ The Research Center for Hepatitis and Immunology National Center for Global Health and Medicine Ichikawa Japan

**Keywords:** biostatistics and bioinformatics, chemotherapy, disease control, hepatitis B virus, infection, reactivation

## Abstract

The risk of hepatitis B virus (HBV) reactivation has increased owing to advances in the immunosuppressive therapy field. However, the HBV reactivation incidence among patients with previously resolved HBV (prHBV) infection during immunosuppressive therapy for rheumatoid arthritis (RA) remains unclear. The objective of this work is to describe the validity of detecting prHBV infection from administrative data through comparisons with chart abstraction and determine the incidence of HBV reactivation during immunosuppressive therapy for RA in Japan. In this retrospective cohort study, data on selected patients were extracted from administrative claims data. To identify patients with prHBV infection and de novo hepatitis, and HBsAg carriers, we conducted chart abstraction. The incidence rate of de novo hepatitis was 1.23 of 100 person‐years. The positive predictive value (PPV) and its 95% confidence interval (CI) of administrative data for the identification of suspected prHBV infections was 85.8% (95% CI: 81.7%‐89.3%). This study evaluated the PPV of the algorithm of HBV‐DNA testing with immunosuppressive therapy performed four times or more per year for the detection of prHBV infection from administrative data. Additionally, we determined the incidence rate of HBV reactivation among preHBV infections during immunosuppressive therapy for RA to be 1.23 of 100 person‐years.

## 1 INTRODUCTION

The risk of hepatitis B virus (HBV) reactivation has increased owing to advances in immunosuppressive therapy.^1^, ^2^ In Japan, 23.2% of blood donors show HBcAb and/or HBsAb‐positivity,^1^ and HBV reactivation due to immunosuppressive medication use is responsible for 6.8% of all fulminant hepatitis cases.^3^ The reactivation of prHBV infection is referred to as de novo hepatitis.

New immunosuppressive therapies have been approved by the Japanese government for entry into the market. Several types have been approved for rheumatic disease treatment. Regarding the risk of HBV reactivation associated with the use of these agents, Japan's Pharmaceuticals and Medical Devices Agency^4^ issued an alert after several patients died of hepatic failure due to de novo hepatitis. The Japan College of Rheumatology, in collaboration with the Japan Society of Hepatology, published “A proposal for management of rheumatic disease patients with HBV infection receiving immunosuppressive therapy” in 2011,^5^ according to which, HBV carriers and previously resolved hepatitis B virus (prHBV) infection patients should be screened before immunosuppressive therapy or chemotherapy. If patients are HBsAg positive, the administration of nucleoside analogues (NAs) is recommended. The HBV‐DNA levels of patients with prHBV infection should be monitored using real‐time polymerase chain reaction every 1 to 3 months. If HBV‐DNA levels exceed 2.1 log copies/mL (20 IU/mL) during monitoring, pre‐emptive NA therapy should be initiated immediately.

However, the incidence of HBV reactivation among patients with prHBV infection during immunosuppressive therapy for rheumatoid arthritis (RA) is still unclear. Previous cohort studies on the incidence of reactivation were conducted in limited populations.^2^ Nowadays, administrative data‐based surveys are being conducted for the investigation of the incidences of different diseases.^6^, ^7^, ^8^ One study that evaluated the incidence and risk factors of de novo used the National Database of Health Insurance Claims and Specific Health Checkups of Japan, in which insurance claim data are comprehensively collected.^9^ This study utilized an enormous dataset to detect a rare event, but it had potential limitations. Analyzing the incidence of HBV reactivation among prHBV infection patients using an administrative data set is challenging, as the identification of prHBV infection and HBV reactivation is difficult. There are no diagnostic codes for these two conditions.

We aimed to describe the validity of detecting prHBV infection using administrative data through comparisons with chart abstraction because the information of medical charts and database under our organization can be linked to each other, and that allows us to determine the incidence of HBV reactivation during immunosuppressive therapy for RA across Japan.

## 2 MATERIALS AND METHODS

### 2.1 Study setting

This was a retrospective cohort study. As of April 2018, in the National Hospital Organization (NHO), established in April 2004, there were 141 hospitals including both general acute‐care hospitals and specialized long‐term care hospitals. All NHO hospitals provide administrative claims data to the Medical Information Analysis (MIA) databank managed by the Clinical Research Center at the NHO Headquarters. The Hepatic Disease Network is one of the clinical networks in the NHO. Four hospitals from this network were included in this study. We chose three hospitals from the western region of Japan and one from the south of Tokyo, as HBV infections are commonly observed in these regions.

### 2.2 Data extraction

Data on adult RA patients, who underwent periodic HBV‐DNA testing between 1 April, 2011 and 31 March, 2015, were extracted from the MIA. RA was defined by the following International Classification of Diseases, 10th Revision (ICD‐10) codes: M059$, M060$, M068$, and M069$. In accordance with the JSH guideline, periodic HBV‐DNA testing was defined as testing that was performed four times or more per year. We excluded patients with human immunodeficiency virus (HIV) infection. We hypothesized that the performance of periodic HBV‐DNA testing during immunosuppressive agent administration is indicative of the presence of prHBV infection. Specialist doctors of the Hepatic Disease Network working for the four enrolled NHO hospitals were assumed to have good adherence to the guideline. Using data obtained from the information system of each hospital, patients were divided into those receiving immunosuppressive therapy and those who did not. Immunosuppressive agents including methotrexate, leflunomide, tofacitinib, tacrolimus, mizoribine, azathioprine, infliximab, etanercept, tocilizumab, adalimumab, abatacept, golimumab, certolizumab pegol, rituximab, ustekinumab, and secukinumab were usually prescribed for outpatients. In addition, patients prescribed with NAs were stratified by the timing of NA prescription: before immunosuppressive therapy, after immunosuppressive therapy, and on the same month as the immunosuppressive therapy.

### 2.3 Chart abstraction

To identify patients with prHBV infection and de novo hepatitis, and HBsAg carriers, physicians (HY, NI) and a pharmacist (SI) conducted chart abstraction of the patients identified through extraction from the MIA and four hospital information systems. Chart abstraction was conducted using current literature and clinical experience, considering the results of blood testing, patient age, sex, diagnosis, use of medications, and referrals. The prHBV and non‐prHBV infection (HBsAg carriers and others) cases were detected through diagnostic testing results. Patients were also detected through medical history checks and referrals from other institutions. For example, patients with serum HBsAg negativity and serum HbsAb and/or HBcAb positivity were defined as having prHBV infection, while some were defined as HBsAg carriers if this was indicated in a letter from other general practitioners. Additionally, reviewers identified de novo hepatitis by a chart review. An HBV‐DNA testing value ≥ 2.1 log copies (20 IU/mL) was used as the cut off; however, the decisions of the physicians who performed the diagnoses were prioritized.

### 2.4 Statistical analysis

We calculated the descriptive statistics of the patient characteristics including mean (standard deviation) for continuous variables and proportions for categorical variables. The positive predictive value (PPV) and its exact 95% confidence interval (CI) of administrative data for the identification of suspected prHBV infection were calculated, considering the chart abstraction result as the reference standard. Using chart abstraction, the annual de novo incidence rates were calculated using the number of de novo hepatitis cases as the numerator and the number of prHBV person‐years as the denominator, were determined during the research period. In patients with NA prescription, the chart abstraction results were stratified by the timing of NA prescription in relationship with immunosuppressive therapy. All analyses were performed using SAS 9.4 statistical software (SAS Institute Inc, Cary, NC).

### 2.5 Ethical approval

The study protocol was approved by the Central Ethics Review Board of the NHO, which waived the need for informed consent from participants (H30‐0611039). The study and the opportunity for participants to opt out of it were announced through the website.

## 3 RESULTS

### 3.1 Patient identification and the incidence rate of de novo hepatitis

**Figure 1:**
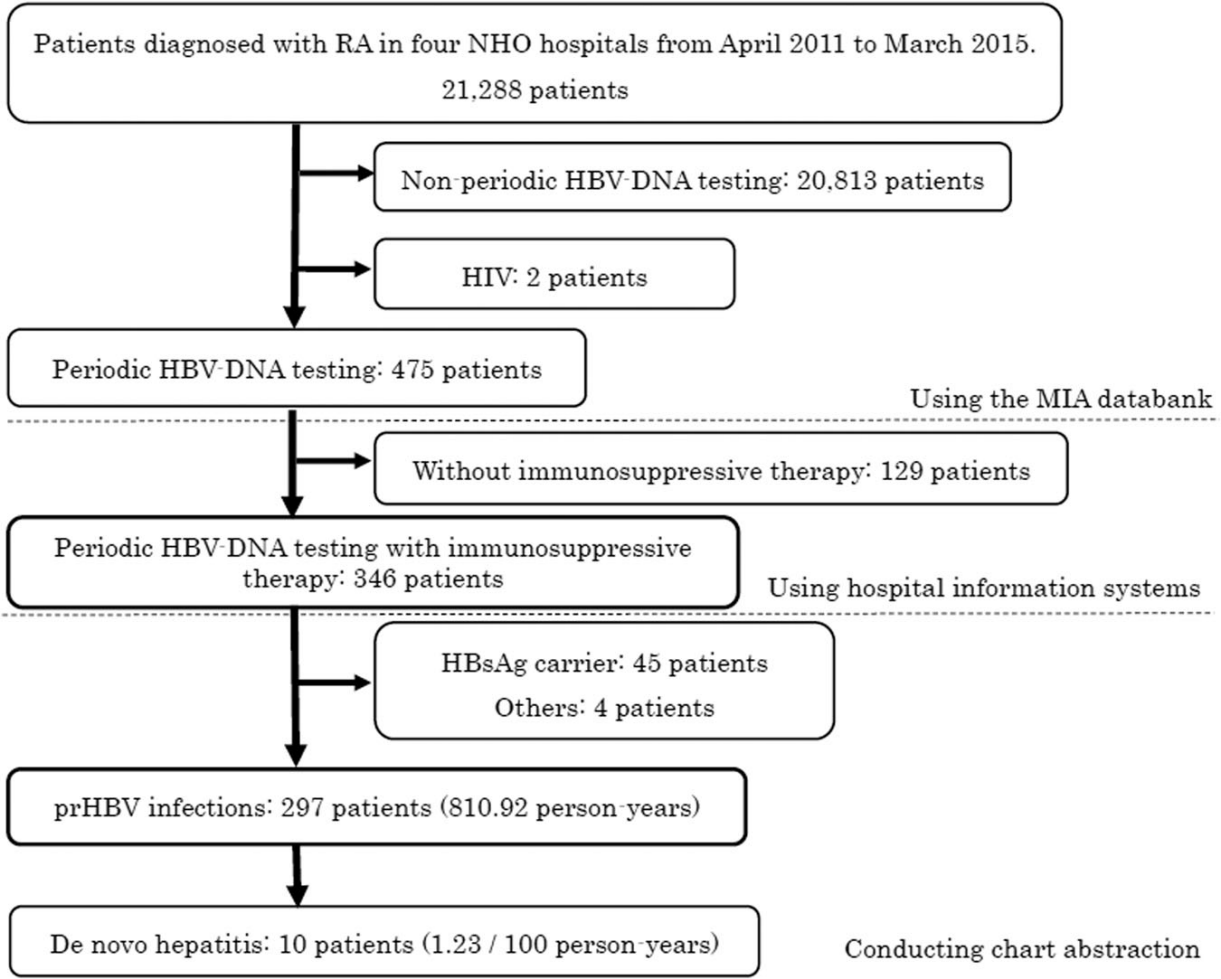
Identification of prHBV infection cases and incidence rate of de novo hepatitis. HBsAg carrier, serum HBs antigen‐positive patients; HIV, human immunodeficiency virus; MIA, Medical Information Analysis; NHO, National Hospital Organization; periodic HBV‐DNA testing, HBV‐DNA testing four times or more per year; prHBV, previously resolved hepatitis B virus; RA, rheumatoid arthritis

Figure 1 shows the proportion of patients with prHBV infections and the incidence rate of de novo hepatitis. A total of 21 288 patients were diagnosed with RA in the four NHO hospitals during the study period. After the exclusion of two HIV‐positive patients, 475 patients were identified using the MIA databank, who had undergone HBV‐DNA testing four times or more per year. Using data from the hospital information systems in the four hospitals, 346 patients were detected as having an immunosuppressive agent prescription. Of the 346 patients, 297 prHBV infection cases, 45 HBsAg carriers, and four others were detected through chart abstraction. The 297 patients showed a cumulative incidence of 810.92 person‐years, through the calculation of the sum of the periods between the day of the first prescription and the day of incidence or the last visit during the research period, then 10 de novo hepatitis cases were detected. The incidence rate of de novo hepatitis during immunosuppressive therapy among the prHBV cases was 1.23/100 person‐years. The characteristics of the 346 patients who underwent chart abstraction are indicated in Table 1. The proportion of female patients was 71.4% (n = 247), while 329 patients (95.1%) had connective tissue disease and/or rheumatic disease, and 139 (40.2%) had mild liver disease. There were 100 patients (28.9%) with peptic ulcer disease and 84 patients (24.3%) had chronic pulmonary disease.

**Table 1:** Characteristics of patients in whom chart abstraction was conducted

	Patients (n = 346)
Age, y (±SD)	63.6 (±10.1)
Female sex, n (%)	247 (71.4%)
Comorbidity, n (%)	
Myocardial infarction	1 (0.9%)
Cerebrovascular disease	29 (8.4%)
Congestive heart failure	40 (11.6%)
Connective tissue disease‐rheumatic disease	329 (95.1%)
Dementia	2 (0.6%)
Diabetes without complications	42 (12.1%)
Mild liver disease	139 (40.2%)
Peptic ulcer disease	100 (28.9%)
Peripheral vascular disease	27 (7.8%)
Chronic pulmonary disease	84 (24.3%)
Cancer	59 (17.1%)
Diabetes with complications	14 (4.1%)
Paraplegia and hemiplegia	4 (1.2%)
Renal disease	14 (4.1%)
Metastatic carcinoma	8 (2.3%)
Moderate or severe liver disease	1 (0.3%)

### 3.2 Validity of the identification of suspected prHBV infection cases and NA use

By conducting the chart abstractions, 297 prHBV cases and 49 non‐prHBV cases were detected among 346 identified as prHBV infections from administrative data. Thence, the PPV and its exact 95% CI of administrative data for the identification of suspected prHBV infection was 85.8% (95% CI: 81.7%‐89.3%), considering the chart abstraction result as the reference standard (Table 2). Of the 346 patients, 49 (14.2%) were prescribed with NAs during the study period. Table 3 shows the various NA and immunosuppressive agent prescription situations. We assumed that 17 patients prescribed with NAs before immunosuppressive therapy were HBV carriers, and 7 patients prescribed with NAs with immunosuppressive therapy in the same month were HBV carriers as well. As a result of chart abstraction, 16 of 17 patients (94.1%) and 6 of 7 patients (85.7%) were HBV carriers, respectively. The 25 patients prescribed with NAs after immunosuppressive therapy were assumed to be de novo infections. However, 16 of 25 patients (64.0%) were HBV carriers.

**Table 2:** Validation of administrative data against hospital chart data

Administrative data	Chart abstraction		N	PPV	95% CI
	prHBV	Non‐prHBV			
Suspected prHBV	297	49	346	85.8%	81.7%‐89.3%

*Note*: Data are n.

Abbreviations: CI, confidence interval; PPV, positive predictive value; prHBV, previously resolved hepatitis B virus; suspected prHBV, HBV‐DNA testing with immunosuppressive therapy performed four times or more per year

**Table 3:** Chart abstraction result by the prescription timing of nucleoside analogue and immunosuppressive therapy

	N	prHBV	HBsAg carrier	Others
		n = 297	n = 45	n = 4
NAs prescription total, n (%)	49 (100%)	9 (18.4%)	38 (77.6%)	2 (4.1%)
NAs prescription before immunosuppressive therapy, n (%)	17 (100%)	1 (5.9%)	16 (94.1%)	0 (0%)
NAs prescription with immunosuppressive therapy in the same month, n (%)	7 (100%)	0 (0%)	6 (85.7%)	1 (14.3%)
NAs prescription after immunosuppressive therapy, n (%)	25 (100%)	8 (32.0%)	16 (64.0%)	1 (4.0%)

Abbreviations: HBsAg carrier, serum HBs antigen‐positive patients; NA, nucleoside analogue; prHBV, previously resolved hepatitis B virus

## 4 DISCUSSION

This study evaluated the PPV of the algorithm for the performance of HBV‐DNA testing four times or more per year and immunosuppressive agent administration in the detection of prHBV infection from administrative data (85.8%; 95% Cl: 81.7%‐89.3%). Additionally, we determined the incidence rate of HBV reactivation among the preHBV infection cases during immunosuppressive therapy for RA in the four NHO hospitals (1.23/100 person‐years). The main concerns when using administrative health data for epidemiological studies are the correct identification of diagnoses and the determination of their validity. Diagnostic codes are commonly used as tracers for diseases.^10^, ^11^ According to the clinical guideline pertaining to the monitoring of HBV‐DNA testing, the present study detected prHBV infections without diagnostic codes due to the rough prHBV infection definition. The high PPV percentage indicates that the physicians working at the NHO hospitals showed high adherence to the clinical guideline.

Although we included study patients simply through administrative data, our result on the incidence rate of de novo hepatitis is similar to that observed in a previous study. Of the 1042 patients in that study, HBV‐DNA was detected in 35 (1.93/100 person‐years).^12^ In that study, physicians from 16 Japanese hospitals enrolled patients and observed their information every time a patient visited. While this method may ensure good quality, there are some issues pertaining to the study size and follow‐up.

For the detection of a small number of de novo hepatitis cases using a large database, HBV carriers were excluded even in the absence of diagnostic codes. A previous study assumed that patients who had been prescribed NAs with immunosuppressive therapy in the same month were HBsAg carriers. However, our data indicated that 16 patients (64.0%) who had been prescribed NAs after immunosuppressive therapy were HBsAg carriers.

The de novo incidence rate may be higher among carriers who were vulnerable to reactivation during immunosuppressive therapy. It might be easy to get contamination when we study without administrative data including blood testing results. A total of 49 carriers were included when we appropriated our algorithm to extract prHBV infection cases. In this study, we were unable to exclude carriers precisely.

The present study has some limitations. First, we did not evaluate the validity of the RA patients' diagnoses. According to our result, 95.1% (329 of 346) of the patients had connective tissue disease and/or rheumatic disease. Moreover, we had already validated the diagnoses with the MIA database in a previous study.^8^ As our study included data from only four NHO hospitals, the generalizability of our findings are limited. However, this was a sample study, and there is potential for the performance of a larger study.

A strength of this study was the performance of chart abstraction. Only NHO hospitals can create a link between the database and clinical charts at the patient level in Japan. The present study used hospital information systems for the identification of immunosuppressive agent prescriptions. However, we can extract those subjects from March 2016. We plan to conduct a study on this topic in the future with the use of administrative data as well as electrical medical record data. That study will be larger in scale, allowing for the easy detection of HBV reactivation incidence during immunosuppressive therapy; our technique for the estimation of this incidence rate may be extended to other diseases too.

## CONFLICT OF INTERESTS

The authors declare that there are no conflict of interests.
